# Individualized anticoagulation monitoring in critically ill patients: study protocol for a prospective observational comparison of Anti-Xa, viscoelastic testing, and thrombin generation

**DOI:** 10.3389/fmed.2026.1758025

**Published:** 2026-04-15

**Authors:** Martin Mirus, Sebastian Scholz, Adrian Schmidt, Paul Leon Petrick, Oliver Grottke, Peter Spieth, Lars Heubner

**Affiliations:** 1 Department of Anaesthesiology and Intensive Care Medicine, Faculty of Medicine and University Hospital Carl Gustav Carus, TUD Dresden University of Technology, Dresden, Germany; 2 Department of Anaesthesiology, RWTH Aachen University Hospital, Aachen, Germany

**Keywords:** anticoagulation, Anti-Xa, critically ill, haemostasis, LMWH, sepsis, UFH, viscoelastic testing

## Abstract

**Introduction:**

Critically ill patients experience high rates of thromboembolic and bleeding events despite the use of pharmacological thromboprophylaxis or weight-based anticoagulation. Laboratory assays for drug monitoring, as Anti-factor Xa (Anti-Xa), overcome the limitations of activated partial thromboplastin time (aPTT) but only quantify drug effects and do not reflect global haemostasis. Viscoelastic testing (VET) may provide additional functional information. Robust clinical data in the ICU setting are limited.

**Objective:**

Primary objective is to assess the association between VET clotting time (CT) in Russell viper venom (RVV) assay and Anti-Xa activity. Secondary objectives include assessing whether VET or Anti-Xa aligns more closely with global haemostatic function, defined by thrombin generation assay (TGA) variables. Exploratory analyses aim to characterise associations among VET parameters, standard laboratory tests, and biomarkers; model dose–exposure–response relationships; and identify clinical modifiers as sepsis or renal dysfunction. Sepsis status will be incorporated as predefined effect modifier in mixed-effects models.

**Study design:**

This single-center, prospective, observational cohort study enrols critically ill adults receiving unfractionated heparin (UFH) or low-molecular-weight heparin (LMWH). Sampling is standardized to pharmacokinetics: 4 h post-dose for subcutaneous LMWH and 4–6 h post-start for i.v. UFH. At each time point, Anti-Xa, VET on ClotPro® (RVV, EX, and RVH assays), standard laboratory tests, and advanced haemostatic markers (factor VIII, factor XIII, PF1 + 2, TGA, PAP, PAI, TFPI, TAT) are collected. Two repeated measurements per patient capture intra-individual variability.

**Expected results:**

We hypothesize that CT-RVV demonstrates a clinically interpretable monotonic relationship with Anti-Xa activity under ICU conditions. We further anticipate discordance between Anti-Xa and global haemostatic markers in a relevant subset of patients, particularly in sepsis, supporting the hypothesis that VET aligns more closely with functional haemostasis than Anti-Xa alone. These findings are expected to delineate when fixed dosing is insufficient, identify predictors of under- or over-anticoagulation, and guide personalised anticoagulation strategies. If RVV-CT consistently correlates with Anti-Xa across clinical states, VET could streamline monitoring where laboratory turnaround time limits timely dose adjustment; if not, the results will clarify the boundaries of VET applicability and highlight scenarios requiring laboratory confirmation or TGA.

**Registration:**

Ethics approval was obtained from the responsible committee of Technical University Dresden (BO-EK-338082024). The study is registered with the German Clinical Trials Register (DRKS00037385), registration date: 10 July 2025, https://drks.de/search/de/trial/DRKS00037385.

## 1 Introduction

Thromboembolic events remain a major cause of morbidity and mortality in critically ill patients (1–4). Despite pharmacological prophylaxis, 5–15% of patients in intensive care units (ICUs) develop deep vein thrombosis (DVT), which is associated with prolonged mechanical ventilation and extended ICU and hospital stays (5–8). Low-molecular-weight heparins (LMWHs) are preferred over unfractionated heparin (UFH) because of their greater antithrombotic efficacy and lower complication rates (9, 10). Optimal anticoagulation requires the risk of thrombosis and bleeding to be balanced. Although LMWHs are often assumed to have predictable pharmacokinetics permitting fixed dosing without monitoring, this assumption does not apply in critical illness. The reported therapeutic failure rates range from 12 to 20%, underscoring the potential value of individualized anticoagulation guided by therapeutic drug monitoring (TDM) (2, 4, 11). Prospective studies have demonstrated wide interindividual variability in Anti-Xa activity despite standardized dosing, largely due to sepsis, multiorgan failure, endothelial dysfunction, and fluid shifts (12, 13). A systematic review of 18 studies including 1,644 ICU patients reported low peak Anti-Xa levels ranging from 0.1 to 0.35 IU/mL, although no consistent association with bleeding was revealed (14). These results indicate that fixed dosing may result in both under- and overtreatment (15–17). Anti-Xa monitoring is already recommended for specific comorbidities, such as obesity and renal impairment (1, 10), but its routine use in ICUs is limited by logistical barriers and turnaround times. Moreover, while Anti-Xa accurately reflects drug concentration, it does not capture the global haemostatic status, which may be profoundly altered in critical illness (18). Standard laboratory tests (SLTs), such as aPTT, fibrinogen, and INR, correlate poorly with anticoagulant effects in ICU patients and are influenced by factors unrelated to heparin activity, such as elevated factor VIII, resulting in misleading results (18, 19). Anti-Xa measurement directly quantifies the pharmacodynamic effects of UFH and LMWH and is considered the reference standard (1, 10, 19), but it remains an incomplete surrogate for haemostatic balance. Viscoelastic testing (VET) offers functional, point-of-care (POC) assessment of coagulation, integrating plasmatic and cellular components in real time. Devices such as TEG6s®, ROTEM®, and ClotPro® measure clot initiation, propagation, firmness, and fibrinolysis in whole blood (20, 21). Several studies have attempted to correlate VET variables with Anti-Xa activity, with mixed results. *In vitro* experiments using TEG6s® demonstrated strong correlations between clotting time (CT) and heparin concentration, with classification accuracies >90% for UFH and >78% for LMWH (22). Healthy volunteer studies have shown a strong correlation between TEG® reaction time and Anti-Xa (*r* = 0.82) and improved prediction using composite variables (23). Modified ROTEM® assays demonstrated near-linear relationships between Anti-Xa and CT (*r* ≈ 0.93) in spiking experiments (24). However, the clinical data are inconsistent: some studies reported no correlation between TEG® reaction time and Anti-Xa beyond single peak levels after LMWH dosing (25); others reported that conventional ROTEM® variables failed to detect prophylactic LMWH effects (26), whereas modified ClotPro® assays showed improved sensitivity (27). Overall, the sensitivity of LMWH to VET appears to be highly context- and assay-dependent (28). Thrombin-generation assays (TGAs) provide a complementary global evaluation by quantifying thrombin kinetics and overall generation. Although their use is hampered by limited standardization, automated TGA platforms have demonstrated good correlation with Anti-Xa at therapeutic LMWH doses and may detect reduced thrombin generation in suspected heparin resistance (29). In critically ill children, the endogenous thrombin potential correlated moderately with the TEG® kinetic time and more strongly with Anti-Xa during extracorporeal support (30). These findings emphasize that Anti-Xa, VET, and TGA capture distinct but overlapping aspects of haemostasis: Anti-Xa reflects drug concentration, whereas VET and TGA integrate functional contributions from coagulation factors, fibrinogen, and platelets (31).

In summary, while Anti-Xa measurement is considered the gold standard for monitoring heparins, its predictive value for adequate prophylaxis or therapy in critically ill patients is uncertain. VET and TGA offer broader insights into haemostasis but yield inconsistent results, particularly at low LMWH doses, and remain poorly standardized. Major gaps therefore persist: fixed dosing yields unpredictable Anti-Xa levels; validated therapeutic and prophylactic targets are lacking; the relationship between Anti-Xa and global haemostatic function is unresolved; and the interplay among dosing, Anti-Xa activity, VET, TGA, and patient-specific factors has not been systematically evaluated.

The present protocol addresses these gaps through a prospective observational study in critically ill adults receiving UFH or LMWH. By concurrently measuring Anti-Xa, viscoelastic variables, thrombin generation, and standard laboratory tests, this study aims to determine whether the VET can serve as a reliable surrogate for Anti-Xa monitoring to obtain a broader view of the coagulation system in critically ill patients.

Because Anti-Xa quantifies UFH and LMWH activity in plasma whereas VET reflects whole-blood clot formation influenced by both anticoagulant exposure and the patient’s haemostatic substrate, a strong correlation is not guaranteed in critical illness. The primary objective is therefore pragmatic: to determine whether CT in RVV assay shows a sufficiently stable, monotonic relationship with Anti-Xa to support bedside interpretation as a rapid surrogate for Anti-Xa activity, and to define possible conditions under which this relationship worsens. A stable correlation would support CT-guided interpretation when Anti-Xa results are delayed or logistically constrained; conversely, weak or heterogeneous correlation would question whether VET-based monitoring offers advantages and underscore the need for complementary testing. Associations with clinical outcomes are collected as exploratory signals; the study is not powered to rank assays by clinical endpoints.

## 2 Methods and analysis

This trial protocol adheres to the SPIRIT guidance for protocols of clinical trials (32).

### 2.1 Trial objectives

*Primary objective:* To assess the association between VET and Anti-Xa in critically ill patients with and without sepsis receiving UFH or LMWH, and to evaluate whether VET can serve as a surrogate for Anti-Xa as the reference assay.

*Secondary objective:* To determine whether VET provides complementary or superior information to Anti-Xa activity for characterizing global haemostatic function, as assessed TGA variables, in critically ill patients with and without sepsis.

*Exploratory objective:* To investigate whether extended VET variables and advanced haemostatic biomarkers provide additional information on anticoagulant effects, to explore dose-exposure-response relationships between drug dose, Anti-Xa, VET, and TGA, to assess the influence of patient-level modifiers, and to generate preliminary signals on the predictive value of laboratory profiles for bleeding and thrombotic complications in critically ill patients with and without sepsis.

Given the observational design and sample size, this study is primarily hypothesis-generating. Clinical events, bleeding and thrombosis, are assessed exploratorily and will be interpreted descriptively without claims of definitive prognostic performance.

### 2.2 Trial design

To address the study objectives, this single-center, prospective, observational cohort study will analyse blood samples collected at two time points during the ICU stay of critically ill patients admitted to the Department of Anaesthesiology and Intensive Care Medicine at the University Hospital Dresden, Germany. The first blood sample is obtained at study inclusion. The second sample is collected 24–72 h later. Sampling will be timed to coincide with the expected peak plasma concentration of the administered anticoagulant. Blood sampling for LMWH will be performed 4 h after the last subcutaneous administration to target peak plasma concentration. Sampling will preferentially be obtained from an indwelling arterial catheter using a standardized discard protocol to minimise contamination. For UFH continuous infusion, sampling will occur at steady state (≥4–6 h after initiation or dose adjustment). At each sampling, the following assessments will be performed:

- Determination of Anti-Xa activity
- VET assays
- Advanced coagulation variables
- SLTs

**Figure 1:**
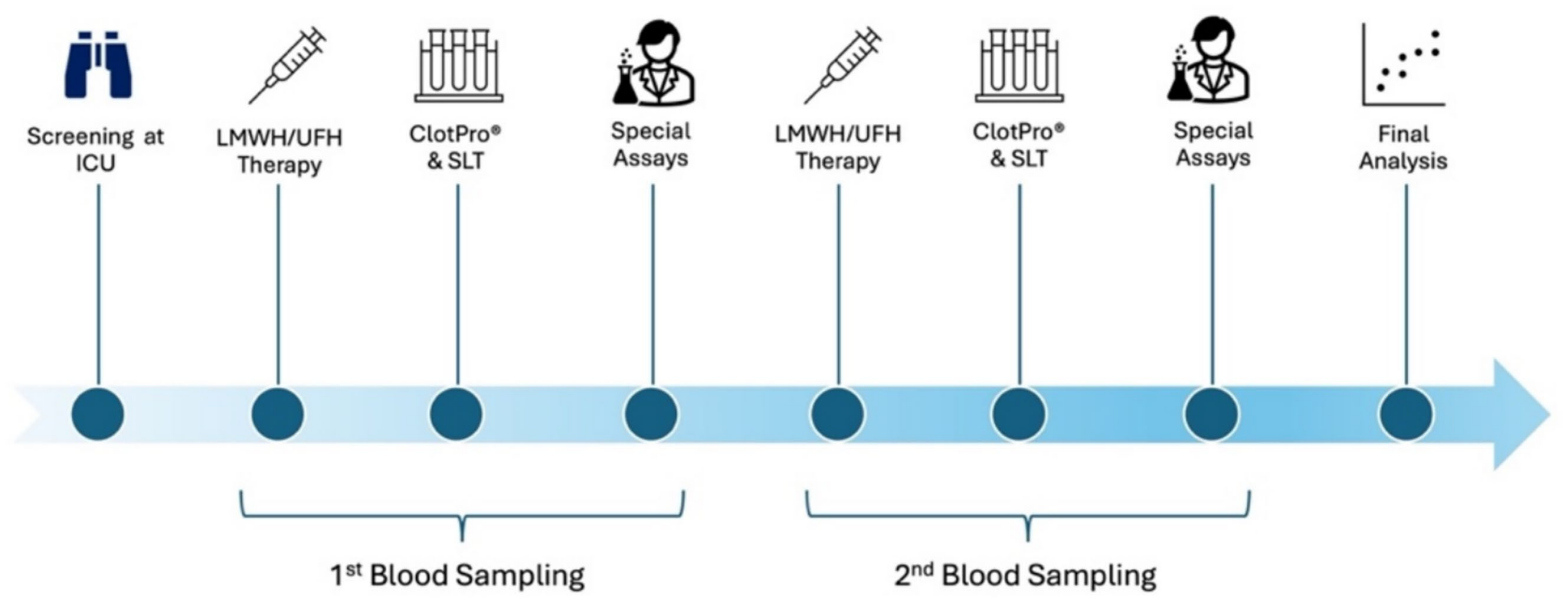
Study flow and haemostatic assessments in trial. Critically ill patients receiving UFH or LMWH are screened and enrolled consecutively. Two blood samples are obtained per patient: the first at study inclusion and the second 24–72 h later. For LMWH, sampling targets peak plasma concentration at 4 h after subcutaneous administration. For continuous UFH infusion, sampling is performed at steady state, defined as ≥6 h after initiation or ≥4–6 h after dose adjustment. Laboratory analyses include viscoelastic testing (ClotPro® RVV, EX, ECA, RVH ± IN/HI), chromogenic Anti-Xa activity (UFH/LMWH), standard laboratory assays (aPTT, INR, antithrombin, fibrinogen, platelet count, D-dimers), and advanced haemostatic markers (factor VIII, factor XIII, PF1 + 2, TGA, PAP, PAI, TFPI, TAT). All assays are derived from the same sampling time point. Plasma samples for thrombin generation and ELISA-based assays are processed immediately, aliquoted, and stored at −80 °C. These measurements are performed in batch analysis to minimise inter-assay variability. aPTT, activated partial thromboplastin time; ECA, ecarin test; INR, international normalized ratio; LMWH, low-molecular-weight heparin; PAP, plasmin-antiplasmin complexes; PAI, plasminogen activator inhibitor; RVV, Russell’s viper venom test; TGA, thrombin generation assay; TAT, thrombin-antithrombin complexes; TFPI, tissue factor pathway inhibitor; UFH, unfractionated heparin; VET, viscoelastic testing.

The Anti-Xa level at the time of sampling reflects the plasma concentration of the anticoagulant and allows consideration of individual variations in drug absorption and elimination. Interpretation of Anti-Xa activity will account for concomitant antithrombin levels, as assay results depend on endogenous antithrombin when no supplementation is included in the reagent, and may be influenced by reagent composition and acute-phase proteins, particularly in patients receiving UFH. For example, high catecholamine requirements or microcirculatory disturbances, such as those occurring during sepsis, may impair the absorption of subcutaneously administered drugs, leading to subtherapeutic plasma levels and necessitating dose escalation. Conversely, reduced drug elimination, such as in renal failure, may cause accumulation and supratherapeutic levels. To account for the specific haemostatic alterations seen in sepsis, sepsis-specific scores will be recorded. Additional laboratory analyses will include prothrombin fragments 1 + 2 (PF1 + 2), thrombin generation assay (TGA), plasmin-antiplasmin complexes (PAP), plasminogen activator inhibitor (PAI), tissue factor pathway inhibitor (TFPI), and thrombin-antithrombin complexes (TAT) to detect hypercoagulable states and/or impaired fibrinolytic activity. SLTs will also be performed to improve comparability among the heterogenous ICU population and to enable subgroup analyses. The anticoagulants of interest are LMWH and UFH. VET testing will be performed using the RVV-test for LMWH and UFH. Both groups will additionally undergo measurements via extrinsic (EX) and Russell viper venom with heparinase (RVH) tests. Measurements will be performed in patients with prophylactic, intermediate, and therapeutic anticoagulation targets, allowing comparisons of the correlation and adjustability of individualized anticoagulation across different degrees of anticoagulation intensity. Figure 1 provides an overview of the temporal sequence of measurements and planned analyses in the study.

### 2.3 Study population and recruitment

Participant selection is based on predefined inclusion criteria and is conducted entirely on a voluntary basis, with informed consent obtained before participation. Given the biological and clinical heterogeneity inherent to critically ill ICU patients, sufficient sample size is required to allow modelling of patient-level variability and pre-specified effect modification.

Inclusion criteria for critically ill patients are as follows:

- ≥18 years
- Undergoing anticoagulation therapy with UFH, LMWH at any dosage
- Written informed consent by patient or legal representative

The following variables are recorded to enable further classification:

- Sepsis or septic shock within the last 48 h according to the SEPSIS-3 criteria (33) using the SOFA score, continuously collected hemodynamic variables and results of blood gas analysis

Exclusion criteria:

- Known hereditary coagulation disorder
- Patients receiving ongoing therapeutic oral anticoagulants (direct oral anticoagulants or vitamin K antagonists) will be excluded from the primary analysis. Patients with prior exposure may be included only if clinically relevant residual drug activity can be excluded based on documented timing and renal function.

All ICU patients treated with UFH or LMWH will be screened, and eligible patients will be consecutively enrolled until the target sample size is reached. Enrolment will be based on fulfilment of the inclusion criteria and will proceed only after fully voluntary informed consent has been obtained. Sepsis status will be defined according to Sepsis-3 criteria (33). SOFA score will be recorded as a continuous variable and additionally explored as a predefined severity modifier (e.g., SOFA ≥ 9) within effect-modification analyses.

### 2.4 Setting and intervention

The study will be conducted at the Department of Anaesthesiology and Intensive Care Medicine, University Hospital Carl Gustav Carus, Technische Universität Dresden, Germany. The department provides tertiary care for critically ill patients and includes multiple specialized intensive care units with a broad spectrum of medical and surgical cases. All laboratory analyses, including point-of-care VET ClotPro® and conventional laboratory assays, will be performed within the hospital’s accredited clinical laboratories. Data collection and storage will take place within the secure institutional network of the University Hospital Dresden.

### 2.5 Laboratory procedures and pre-analytical handling

Blood will be drawn from an arterial line using a standardized discard volume. For VET, citrated whole blood (3.2% sodium citrate) will be analysed on ClotPro® immediately after gentle inversion and within 120 min of sampling under controlled temperature conditions as per manufacturer instructions. For plasma-based assays (Anti-Xa, TGA, SLTs, and specialized markers), samples will be collected in 3.2% citrate tubes and processed within 60 min. Plasma preparation will be performed in a designated laboratory area using pre-programmed centrifugation settings. After centrifugation, supernatant plasma will be aliquoted into polypropylene tubes (300 μL per aliquot) and temporarily stored at −80 °C storage for batch analysis. This procedure ensures minimisation of platelet contamination and stabilisation of labile coagulation markers (e.g., PF1 + 2, TAT, TGA parameters).

### 2.6 Standard laboratory tests

Conventional laboratory samples will be transported to the central laboratory. The following assays will be performed: Quick/INR, aPTT, Anti-Xa activity, Antithrombin, D-dimers, fibrinogen, factor VIII (UFH patients only), factor XIII, platelet count, and routine biochemistry (creatinine, urea, estimated GFR, ASAT, ALAT, bilirubin, triglycerides, albumin). Anti-Xa activity will be measured using a chromogenic assay (platform and reagent specified in the final manuscript), with no exogenous antithrombin is added. Antithrombin concentration will be recorded concomitantly. Routine biochemistry and inflammatory markers (CRP, procalcitonin) will only be re-measured if the last determination exceeds 24 h; otherwise, values from the daily clinical laboratory will be used. Routine coagulation parameters (Quick/INR, aPTT, fibrinogen, D-dimers) and Anti-Xa activity will be measured using a STAGO coagulation platform (Diagnostica Stago, France). The chromogenic Anti-Xa assay will be performed according to manufacturer instructions without supplementation of antithrombin.

Routine biochemistry (creatinine, urea, ASAT, ALAT, bilirubin, triglycerides, albumin) and inflammatory markers (CRP, procalcitonin) will be analysed using standard automated hospital laboratory systems. Platelet count will be obtained from standard automated haematology analysers.

Arterial blood gas analysis will provide calcium, haemoglobin, and hematocrit values at the time of sampling.

### 2.7 Viscoelastic testing

Viscoelastic testing will be performed immediately on the ICU using the ClotPro® device. Testing will follow manufacturer-specific standard operating procedures. After electronic patient identification entry, the corresponding test channel and cartridge will be selected. Test-specific pipette tips will be scanned, and citrated whole blood will be transferred into the test cup, gently mixed, and the measurement initiated.

The following assays will be performed according to anticoagulant type:

- LMWH / UFH: EX-, RVV-, RVH-, ECA-test.
- UFH: additional IN/HI-test.

Recorded parameters include CT, CFT, A5, A10, MCF, lysis time (LT), maximum lysis (ML), and alpha angle. Time from sampling to test initiation will be documented. After test completion, measurement channels will be prepared according to hygiene standards. Residual blood will be discarded in designated biohazard containers. The POC devices used in this study, which are operated outside the central laboratory, undergo weekly quality control in accordance with the manufacturer’s specifications.

### 2.8 Thrombin generation

Thrombin generation will be measured using the Innovance® ETP assay (Siemens Healthineers, Marburg, Germany) on an automated coagulation analyser (BCS XP, Siemens Healthineers). Measurements will be performed in platelet-poor plasma. Coagulation will be initiated by addition of tissue factor and phospholipids in the presence of calcium according to manufacturer instructions. Thrombin formation will be quantified via cleavage of a chromogenic substrate, and the increase in optical signal over time will be recorded continuously. The assay software corrects for α2-macroglobulin–bound thrombin activity. From the resulting thrombin generation curve, the following parameters will be derived:

- Endogenous thrombin potential (ETP; area under the curve)
- Peak thrombin generation
- Lag time (time to initiation)
- Time to peak thrombin generation

Thrombin generation is included as a plasma-based global coagulation assay to complement whole-blood viscoelastic testing and Anti-Xa activity measurements. We acknowledge that PPP-based TGA differs from whole-blood clot dynamics and is sensitive to cellular effects; therefore, TGA results will be interpreted as a complementary plasma-based global readout rather than a direct proxy for whole-blood viscoelastic parameters.

### 2.9 Haemostatic markers

The following ELISA-based assays will be used: Plasmin–antiplasmin complexes (PAP): Cloud-Clone Corp.; Plasminogen activator inhibitor-1 (PAI-1): Technozym®; Tissue factor pathway inhibitor (TFPI): Cloud-Clone Corp.; Thrombin–antithrombin complexes (TAT): Cloud-Clone Corp.; Prothrombin fragments 1 + 2 (PF1 + 2): Cloud-Clone Corp.; All assays will be performed according to manufacturer protocols. Batch analysis will be conducted where applicable to reduce inter-assay variability. The ELISA workflow is planned to include the initial assay implementation and also the routine analysis of each sample in duplicate and the inclusion of a standard curve for each assay run as the reference curve.

### 2.10 Obtained laboratory and patient data

Primary outcome

1) Variable: VET CT in the RVV test (UFH/LMWH).
  - Metric: Correlation with Anti-Xa activity (for UFH/LMWH); correlation coefficient (Spearman); multivariable mixed-effects regression to account for repeated measures.
  - Aggregation: Point estimates with 95% CI.
  - The time points are as follows: 4 h after the administration of LMWH (expected peak level) or steady-state sampling during continuous UFH infusion.
  - Clinical relevance: Evaluates whether VET-CT can serve as a surrogate for Anti-Xa and assesses its potential as a surrogate monitoring tool.

Secondary outcomes

1) Variable: VET CT in the RVV test (UFH/LMWH); Anti-Xa activity.
  - Metric: Correlation with TGA variables.
  - Aggregation: Spearman correlation coefficients with 95% CIs and regression coefficients for adjusted analyses.
  - Time point: As the primary outcome.
    Clinical relevance: Examines whether VET provide complementary or superior information to Anti-Xa activity for characterizing global haemostatic function.

Exploratory outcomes

1) Variable: Additional VET variables (CFT, A5, A10, MCF, *α*-angle, lysis time, and lysis index).
  - Metric: Spearman correlations with Anti-Xa and TGA variables; multivariable mixed-effects regression to account for repeated measures.
  - Aggregation: Correlation coefficients (*r*) with 95% CI; regression *β* with 95% CI.
  - Time point: As in primary outcome.
  - Clinical relevance: Explores whether extended VET variables provide complementary or superior information to CT for characterizing anticoagulant effect.
2) Variable: Advanced haemostatic biomarkers (PF1 + 2, TAT, PAP, PAI-1, TFPI, FVIII, FXIII).
  - Metric: Correlation with Anti-Xa, VET, and TGA; exploratory path analyses of marker interrelations.
  - Aggregation: Correlation coefficients with 95% CI; descriptive effect-size comparison.
  - Time point: Parallel to VET and TGA sampling.
  - Clinical relevance: Identifies mechanistic markers that may explain discordance between concentration-based assays (Anti-Xa) and functional assays (VET/TGA).
3) Variable: Dose-exposure-response relationships (applied dose, Anti-Xa levels, VET, and TGA).
  - Metric: Linear and nonlinear regression models linking administered anticoagulant dose to laboratory readouts.
  - Aggregation: Regression coefficients (*β*) with 95% CI; model fit indices (*R*^2^, AIC).
  - Time point: At all scheduled samplings, stratified by regimen type.
  - Clinical relevance: Assesses whether dosing strategies achieve expected anticoagulant activity defined as Anti-Xa activity within the intended target range regarding dosage and whether functional assays reveal discrepancies not captured by Anti-Xa.
4) Variable: Patient-level modifiers (e.g., sepsis status, SOFA score, renal function, albumin, BMI).
  - Metric: Interaction terms in regression/mixed-effects models; subgroup analyses.
  - Aggregation: *β* coefficients for interaction effects with 95% CI; stratified correlation estimates.
  - Time point: As in primary outcome, stratified by clinical subgroup.
  - Clinical relevance: Determines whether specific patient factors systematically influence anticoagulant effect or modify the correlation between Anti-Xa, VET, and TGA.
5) Variable: Clinical complications (bleeding and thrombotic events within 24 h).
  - Metric: Exploratory descriptive analysis of concordance between laboratory profiles and event occurrence; logistic regression if case numbers allow.
  - Aggregation: Odds ratios with 95% CI (if feasible); otherwise descriptive frequencies.
  - Time point: Event assessment in the 24 h prior to or following each sampling.
  - Clinical relevance: Provides preliminary signal on the clinical predictive value of Anti-Xa, VET, and TGA in identifying patients at bleeding or thrombotic risk.

Specialized haemostasis markers are included to mechanistically characterise discordant profiles between concentration-based heparin activity (Anti-Xa) and functional assays (VET/TGA): PF1 + 2 and TAT as thrombin generation/consumption markers; TFPI as a heparin-responsive anticoagulant pathway marker; PAP and PAI-1 as fibrinolysis activity/inhibition markers. These variables will be analysed descriptively and in focused secondary models to reduce data-driven inference. Table 1 provides an overview about the obtained data.

**Table 1:** Collected study variables.

Grouping	Variables
Demographic data	Date of ICU admission; Admission diagnosis; Main diagnosis; Gender; Date of birth; Age; Height; Weight; Date of blood collection
Comorbidities	Trauma; Injury Severity Score; Diabetes; Obesity; Coronary heart disease; Chronic arrhythmia; COPD; Other lung diseases; Venous thrombosis; Nicotine abuse; Active tumour disease; Chronic renal insufficiency; Chronic dialysis; Bleeding disorder upon admission; Thromboembolic complication upon admission; Ischemic stroke; Haemorrhagic stroke; Nosocomial infection; Liver diseases
Condition at time of blood collection	Ventilation status; Glasgow Coma Scale; SpO₂; PaO₂; FiO₂; Systolic blood pressure; Diastolic blood pressure; Mean arterial pressure; Heart rate; Catecholamine use and dosage; Inotrope use and dosage; Temperature; Spontaneous respiratory rate; ECMO status; Date of ECMO initiation
Complications within last 24 h	Bleeding (date, volume, transfusion requirements: RBC, FFP, platelets, PPSB, other blood products); Thrombosis; Pulmonary embolism
Severity scores	SOFA; SAPS II
Medication	Sedation and dosage; Platelet aggregation inhibition and dosage; Oral anticoagulation (drug, dosage, time of last intake); Other anticoagulants and dosage; UFH dosage; LMWH dosage and drug name; Parenteral nutrition; Immunosuppressants
Viscoelastic testing (ClotPro®)	LMWH: EX, RVV, ECA, RVH; UFH: EX, RVV, ECA, RVH, IN, HI; Recorded variables: CT, CFT, A5, A10, MCF, Lysis time, Alpha angle
Standard laboratory tests (SLT)	Haemoglobin; Hematocrit; Platelet count; Leukocytes; Prothrombin time (Quick); aPTT; Anti-Xa; dTT; Antithrombin; D-dimer; Fibrinogen; Calcium; Factor VIII; Factor XIII; C-reactive protein; Procalcitonin; Creatinine; Glomerular filtration rate; Urea; ALAT; ASAT; Bilirubin; Albumin; Triglycerides
Advanced haemostatic assays	TAT; PF1 + 2; TGA; PAP; PAI; TFPI

ALAT, alanine aminotransferase; ASAT, aspartate aminotransferase; aPTT, activated partial thromboplastin time; CFT, clot formation time; CT, clotting time; dTT: diluted Thrombin Time; ECMO, extracorporeal membrane oxygenation; ECA, ecarin test; EX, extrinsic test; FiO₂, fraction of inspired oxygen; FFP, fresh frozen plasma; GFR, glomerular filtration rate; HI, heparinase test; ICU, intensive care unit; IN, intrinsic test; LMWH, low-molecular-weight heparin; MAP, mean arterial pressure; MCF, maximum clot firmness; OAC, oral anticoagulation; PaO₂, arterial partial pressure of oxygen; PAP, plasmin–antiplasmin complex; PAI, plasminogen activator inhibitor; PPSB, prothrombin complex concentrate; Quick, prothrombin time; RBC, red blood cells; RVH, Russell’s viper venom high; RVV, Russell’s viper venom test; SAPS II, Simplified Acute Physiology Score II; SLT, standard laboratory test; SOFA, Sequential Organ Failure Assessment; SpO₂, peripheral oxygen saturation; TAT, thrombin–antithrombin complex; TFPI, tissue factor pathway inhibitor; UFH, unfractionated heparin; vWF, von Willebrand factor.

### 2.11 Timeline

The study is planned to run over a total period of two years, with a recruitment phase lasting 12 months.

### 2.12 Sample size

This study is designed to evaluate the association between Anti-Xa activity and VET variables, with a primary focus on CT in the RVV test, in critically ill patients receiving LMWH or UFH. The planned sample size allows estimation of this association under real-world ICU conditions and supports pre-specified mixed-effects modelling incorporating a limited number of biologically plausible effect modifiers. To account for heterogeneity in coagulation physiology, anticoagulant type (LMWH vs. UFH) and sepsis status will be examined as predefined effect modifiers within the modelling framework. These factors will not be treated as independently powered subgroups but will be analysed using interaction terms. The study is not powered for definitive detection of small correlation coefficients nor for confirmatory subgroup comparisons. Stratified analyses are therefore considered exploratory and hypothesis-generating. Findings will be interpreted cautiously and are intended to inform the design of future adequately powered multicentre studies.

The study is powered for estimation of the Anti-Xa–CT association. Analyses involving thrombin generation are predefined as secondary and exploratory. No separate formal sample size calculation was performed for TGA-CT correlations, as these analyses are hypothesis-generating. The sample size estimation was based on published data reporting a significant Spearman correlation for LMWH-treated patients between Anti-Xa levels and CT-RVV (*r* = 0.669, *p* = 0.0395) (34). For UFH, no exact coefficient was reported, although the same study demonstrated a clear association. For conservative planning, an effect size of *r* = 0.6 was assumed for UFH.

Calculations were performed with G*Power 3.1 (35) for a two-sided test of correlation (*α* = 0.05, power = 0.80). The required sample sizes were 19 independent observation pairs for *r* = 0.669 and 23 pairs for *r* = 0.6. As two temporally separated measurements are planned per patient, the non-independence of repeated measures was adjusted using a design effect with an intraclass correlation coefficient (ICC) of 0.3:


Design−Effect=1+(2−1)×0.3=1.3

$$\text{Effective observations}\;\mathrm{per}\;\text{patient}=\frac{2}{1.3}\approx 1.54$$

Thus, two correlated measurements correspond to approximately 1.54 independent observations. This results in approximately 13 LMWH-treated and 15 UFH-treated patients required to achieve the planned statistical power per anticoagulant category.


$$\mathrm{LMWH}:\frac{19}{1.54}\approx 13\;\text{patients}$$

$$\mathrm{UFH}:\frac{23}{1.54}\approx 15\;\text{patients}$$

Allowing for a 15% dropout rate and to ensure stable estimation within pre-specified mixed-effects models under heterogeneous ICU conditions, the target sample size was set to 30 LMWH-treated and 36 UFH-treated patients (total *n* = 66).

Sepsis status and other clinically relevant variables will be analysed as pre-specified effect modifiers within the mixed-effects framework rather than as separately powered subgroups.

### 2.13 Statistical analysis

All analyses will follow a pre-specified hierarchical statistical analysis plan finalised prior to database lock to minimise the risk of data-driven inference.

#### 2.13.1 Primary analysis

The primary outcome, defined as the correlation between Anti-Xa activity (UFH/LMWH) and CT in the RVV-assay. Because two measurements per patient are planned, the primary inferential analysis will be conducted using a linear mixed-effects model including:

- A random intercept for patient
- A fixed effect for anticoagulant type (UFH vs. LMWH)

This approach accounts for intra-individual correlation and avoids inflation of type I error due to non-independence of repeated observations. Results will be reported as regression slope estimates (per 0.1 IU/mL increase in Anti-Xa activity) with 95% confidence intervals. For descriptive comparability with previous literature, Spearman rank correlation coefficients with bootstrap-derived 95% confidence intervals will additionally be reported:

- For the overall cohort
- Stratified by anticoagulant type

Passing–Bablok regression will be applied as a robust method-comparison technique to assess proportional and systematic bias between CT-RVV and Anti-Xa activity (36). Proportional bias will be considered absent if the 95% confidence interval (CI) of the slope includes 1.0, and constant bias absent if the 95% CI of the intercept includes 0.

These criteria are used for methodological characterization rather than to demonstrate assay equivalence. The primary objective is not interchangeability but to determine whether CT-RVV exhibits a stable monotonic relationship with Anti-Xa activity under ICU conditions.

#### 2.13.2 Pre-specified effect modification analyses

To explicitly address biological heterogeneity in critically ill patients, potential effect modifiers will be analysed within the mixed-effects framework using interaction terms.

Pre-specified modifiers include:

- Sepsis status
- Antithrombin concentration
- Renal function (creatinine, estimated GFR)
- Inflammatory markers (CRP, fibrinogen, albumin)

Sepsis status will be analysed as an effect modifier rather than as a separately powered subgroup.

These analyses aim to identify conditions under which CT-RVV and Anti-Xa activity diverge.

#### 2.13.3 Secondary analysis

To limit multiplicity and reduce type I error inflation, secondary analyses are structured into predefined parameter families.

1. Additional VET Parameters: CFT, A5, A10, MCF, *α*-angle, lysis time, maximum lysis
  - Descriptive Spearman correlations with Anti-Xa
  - Mixed-effects regression models to explore surrogate potential
2. Standard Laboratory Assays: INR, aPTT, fibrinogen, platelet count, antithrombin, bilirubin, triglycerides, albumin
  - Partial correlations
  - Multivariable mixed-effects regression
  - Standardized *β*-coefficients with 95% confidence intervals
  - Change in explained variance (Δ*R*^2^)
3. Advanced Haemostatic Markers: FVIII, FXIII, thrombin generation parameters, PAP, PAI-1, TFPI, TAT
  - Descriptive correlation analyses
  - Comparative interpretation of effect sizes

False discovery rate correction using the Benjamini–Hochberg procedure will be applied within each predefined parameter family.

All baseline characteristics will be summarized descriptively. Categorical variables are reported as counts and percentages. Continuous variables will be summarized as mean ± SD if normally distributed or as median (IQR) if non-normally distributed. Normality will be assessed using the Shapiro–Wilk test.

All analyses will be conducted using SPSS® (version 27, IBM Corp., Armonk, NY, USA) and RStudio (Posit Software, PBC, Boston, USA, version 2025.05.0 + 496). A two-sided *p*-value <0.05 will be considered statistically significant.

### 2.14 Handling for repeated measurements

All inferential analyses involving repeated measurements will apply mixed-effects modelling with patient-level random intercepts. Simple correlation coefficients are presented for descriptive purposes only.

### 2.15 Missing data

Patients with at least one valid paired Anti-Xa and CT measurement will be included in the analysis (modified intention-to-observe population). Missing data will not be imputed. For repeated-measures models, maximum likelihood estimation will be used, which provides unbiased estimates under the assumption of missing at random. Sensitivity analyses will assess the impact of incomplete second measurements.

### 2.16 Additional analyses

Dose–exposure–response relationships between administered anticoagulant dose, Anti-Xa activity, and VET parameters will be explored using linear mixed-effects models. Potential effect modification by patient-specific variables (e.g., age, sex, renal function, SOFA score, SAPS II) will be examined using interaction terms within the mixed-effects framework. No separate subgroup analyses are planned. Stratified analyses, if presented, will be considered descriptive. Exploratory analyses of clinical events (bleeding or thrombotic complications) will use logistic regression only if event numbers permit stable estimation. These analyses are considered hypothesis-generating.

## 3 Discussion

Anti-Xa activity remains the reference standard for monitoring heparin therapy but provides only a narrow perspective on anticoagulant effect. In critically ill patients, fixed dosing frequently results in sub- or supratherapeutic levels, and Anti-Xa values correlate poorly with clinical outcomes. Moreover, logistical barriers and delayed turnaround limit its feasibility as a routine monitoring tool in intensive care. Global assays such as the VET offer broader functional evaluation by integrating plasmatic and cellular components of coagulation. Experimental and volunteer studies demonstrated strong correlations between the VET clotting time and heparin concentration, and modified assays achieved high classification accuracy for therapeutic ranges. However, clinical data are inconsistent: some reports failed to show correlations beyond peak levels or at prophylactic dosing, and sensitivity varied considerably across devices and reagents. Similarly, TGA has shown promise at therapeutic LMWH concentrations but lacks standardization and is less reliable at lower doses. These limitations are particularly relevant in critical illness. Septic patients exhibit profound, dynamically evolving disturbances of haemostasis driven by inflammation, endothelial dysfunction, altered antithrombin levels, and consumption or dysregulation of pro- and anticoagulant pathways. These mechanisms result in substantial variability in heparin pharmacokinetics and pharmacodynamics, making fixed-dose anticoagulation particularly unreliable. In a larger cohort of critically ill COVID-19 patients with repeated paired measurements, CT-RVV showed only a weak-to-moderate correlation with Anti-Xa, highlighting that CT-based detection may be strongly context-dependent (17). This underscores the need to identify biological and methodological modifiers (e.g., inflammation, AT levels, factor consumption) that may explain discordant profiles. Anti-Xa quantifies circulating heparin activity but does not capture the global coagulation phenotype characteristic of sepsis, including heightened thrombin generation, impaired fibrinolysis, or parallel bleeding risk. VET may better reflect this complex state by integrating cellular and plasmatic contributions to clot formation. Although, VET also has clinically relevant limitations in the ICU: sensitivity to platelet dysfunction and mild fibrinolysis is assay- and device-dependent, and acute-phase changes (e.g., elevated fibrinogen) can increase clot firmness and partially mask anticoagulant effects. Nevertheless, these results may better reflect the overall state of the patient’s coagulation system. Therefore, VET is interpreted as a functional whole-blood phenotype that may complement—but not universally replace—concentration-based Anti-Xa monitoring. Understanding the relationship between Anti-Xa, VET, and global haemostatic markers in septic patients is therefore essential to determine whether Anti-Xa alone is sufficient or whether functional assays can identify under- or over-anticoagulation earlier. Given the high incidence of thromboembolic and bleeding complications in sepsis, this study’s findings are expected to directly inform individualized anticoagulation strategies and support more reliable monitoring pathways in this high-risk population.

Sepsis, multi-organ failure, and endothelial dysfunction alter pharmacokinetics and pharmacodynamics, complicating both drug absorption and elimination. Consequently, Anti-Xa monitoring provides only a concentration-based readout and does not reflect the complex interplay between the procoagulant and anticoagulant pathways. In particular, it neglects the influence of elevated fibrinogen levels due to inflammation, increased platelet activation, and impaired fibrinolytic activity. Similarly, standard laboratory tests such as aPTT and INR are confounded by acute-phase reactants and inflammatory mediators. Additionally, Anti-Xa activity reflects heparin-dependent inhibition of a fixed amount of exogenous factor Xa and is therefore largely independent of endogenous factor X levels. In contrast, CT-RVV in whole blood may be influenced by variations in coagulation factor concentrations, including factors V and X. Such variability is considered part of the biological context of critical illness and is not treated as methodological noise but as a potential modifier of assay concordance. As a result, neither Anti-Xa nor conventional coagulation tests adequately capture the dynamic risk of bleeding and thrombosis in critically ill patients.

Point-of-care tests such as VET provide the practical advantage of immediate bedside results, potentially enabling timely adjustment of therapy. As has already been shown, VET can complement the monitoring of argatroban therapy and, in certain situations, may serve as the sole method, providing better performance than aPTT but without exceeding the accuracy of dTT (37). However, robust evidence on whether VET can substitute for or complement Anti-Xa monitoring in UFH and LMWH administration in critically ill patients is still lacking. Consequently, the clinical utility of these assays remains uncertain, particularly in sepsis and at prophylactic LMWH dosing, where therapeutic failure is frequently observed. The present study addresses these gaps by prospectively measuring Anti-Xa, VET, TGA, and extended haemostatic markers in critically ill patients treated with UFH or LMWH. By systematically evaluating correlations, dose–response relationships, and patient-specific modifiers, the trial aims to clarify whether VET clotting times in the RVV test can serve as surrogates for Anti-Xa. In addition, secondary analyses will assess whether VET or Anti-Xa aligns more closely with global haemostatic function, as defined by TGA variables, and whether TGA can help explain discordant results between concentration-based monitoring and functional assays. Ultimately, the results are expected to define the potential and limits of bedside viscoelastic monitoring and to guide patient-tailored anticoagulation strategies aimed at reducing both thrombotic and bleeding complications.

Several practical and operational considerations must be acknowledged when implementing the trial. First, patient recruitment in the ICU setting presents inherent challenges. The study population is characterized by acute illness, rapid clinical deterioration, and a frequent need for urgent interventions. These factors may complicate the timing of informed consent and sample collection and may limit the number of patients eligible for inclusion at predefined sampling points. The sample size was selected to allow estimation of the Anti-Xa–CT association under heterogeneous ICU conditions and to enable modelling of pre-specified effect modifiers. Nevertheless, subgroup analyses remain exploratory. Coordination with treating clinicians will therefore be essential to ensure both feasibility and minimal interference with clinical care. Second, laboratory logistics require careful planning. Anti-Xa assays and the advanced haemostatic variables utilized in this protocol are not available as POC tests and necessitate plasma preparation, specialized reagents, and centralized laboratory support. Turnaround times, sample transport, and pre-analytical variables (e.g., platelet factor 4 release, sample handling under emergency conditions) may affect the results. In contrast, VET devices provide real-time bedside results but require trained personnel, standardized procedures, and rigorous quality control to ensure reproducibility across time points and operators. Ensuring consistent calibration of instruments and adherence to testing protocols is therefore critical. Third, the study must account for the heterogeneity of critically ill patients. Pathophysiological differences, including sepsis, organ dysfunction, and variable pharmacokinetics of anticoagulants, may influence the comparability of the results. This variability is intrinsic to the research question, but it complicates data interpretation and increases the need for a sufficient sample size and stratified analyses to identify relevant subgroups. Fourth, operational constraints in integrating advanced haemostatic assays into routine workflows should be considered. Thrombin generation assays, while promising as global markers of coagulation, are technically demanding, require specialized laboratory infrastructure and are not standardized across platforms. These factors may limit their widespread adoption outside research settings, and interpretation of results must be made with caution.

Finally, ethical and organizational considerations are relevant. Many patients in the ICU lack the capacity to consent, necessitating surrogate decision-making or deferred consent procedures, which must comply with local regulations.

In summary, while this study is designed to answer important clinical questions, it faces challenges related to patient recruitment, laboratory logistics, assay standardization, and the complexity of critical illness. Addressing these operational aspects proactively will be essential for the successful conduct of the trial and for the generation of robust, generalizable results.

## 4 Data management

### 4.1 Documentation

All data collected during the study, including medical history, comorbidities, test results, and adverse events, will be recorded by authorized personnel. A special identification list will be maintained to ensure the identification of patients and participants after the study has been completed. If a participant withdraws consent, they will be removed from the identification list to ensure data privacy and confidentiality.

### 4.2 Data collection

Patient data will be collected via the patient data management system of the intensive care unit of the study center. Data collection will be carried out in accordance with the established study methods, and all collected data will be treated in compliance with data protection regulations. Demographic and clinical variables, including sepsis scores and severity indices, will be documented pseudonymized in standardized case report forms (CRFs). Data collection will follow predefined protocols, and all assessors will be trained in study procedures to ensure reproducibility and data quality.

### 4.3 Withdrawal of individual participants (dropout)

If the exclusion criteria are unknowingly met at the time of study enrolment, later exclusion may occur. Withdrawal of consent will result in the exclusion of the collected data. Published data are untraceable once published.

### 4.4 Data privacy and confidentiality

Data are collected pseudonymously, meaning that identification can only occur via the identification list. The identification list linking study codes to personal identifiers will be stored separately and securely, accessible only to authorized study personnel. No names, initials, or directly identifying data will appear in the study database or publications. Participants can withdraw their consent at any time. The raw data will be stored for at least 10 years in accordance with the “Statute for Ensuring Good Scientific Practice” of the TUD Dresden, Dresden, Germany. All handling of personal data complies with the EU General Data Protection Regulation (GDPR) and institutional policies on data protection.

### 4.5 Archiving of original data

The collected data are stored in an electronic database within the network of the University Hospital Dresden. The data were deleted no earlier than 10 years after the study’s conclusion.

### 4.6 Access to data

The data collected as part of this study will be made available in anonymized form in the Open Access Repository and Archive for Research Data of Saxon Universities OPARA (38).

### 4.7 Methods in analysis to handle protocol non-adherence and any statistical methods to handle missing data

As this is an observational study, no per-protocol versus intention-to-treat analysis distinction applies. The analysis population will include all patients with at least one valid paired measurement of Anti-Xa and VET (modified intention-to-observe). Patients who withdraw consent will be censored at their last available measurement. Missing data will be assessed for this mechanism. All patients with at least one valid measurement will be included in the analysis. For analysis of repeated measures, incomplete data points will be excluded pairwise in correlation analyses. For data considered missing at random, no data imputation is planned.

## 5 Ethics and dissemination

### 5.1 Declaration of Helsinki

The study will be conducted in accordance with the current Declaration of Helsinki and all applicable laws. Ethical approval was obtained from the relevant committee (BO-EK-338082024). The study is registered with the German Clinical Trials Register (DRKS00037385), registration date: 10 July 2025 (39).

### 5.2 Ethics committee

The study protocol was submitted for review to the Ethics Committee of the Technical University of Dresden and was approved before the start of the study (BO-EK-338082024). Any changes to the protocol will be submitted as amendments to the responsible ethics committee.

### 5.3 Participant information and consent

The pseudonymized data collection from clinical routine does not require patient consent according to local law (§ 29 Abs. 1 SaechsKHG). However, additional data collection requires informed consent. Written, informed consent to participate will be obtained from all patients or, in the case of patients lacking capacity, from their legal representatives prior to study enrolment.

### 5.4 Handling of incidental findings

If any abnormal lab results arise in patients, they will be discussed with the treating physicians of the ICU. However, incidental findings in patients are not expected, as most diagnostics are part of routine clinical procedures.
